# Is Silver a Precious Metal for G-Quadruplex Stabilization Mediated by Porphyrins?

**DOI:** 10.3390/ijms252413556

**Published:** 2024-12-18

**Authors:** Nuno M. M. Moura, Sofia Guedes, Diana Salvador, Helena Oliveira, M. Graça P. M. S. Neves, Catarina I. V. Ramos

**Affiliations:** 1 LAQV-REQUIMTE, Department of Chemistry, University of Aveiro, 3810-193 Aveiro, Portugal; nmoura@ua.pt (N.M.M.M.); sguedes@ua.pt (S.G.); 2 CESAM-Centre for Environmental and Marine Studies, Department of Biology and CESAM, University of Aveiro, 3810-193 Aveiro, Portugal; diana.s@ua.pt (D.S.); holiveira@ua.pt (H.O.); 3 CICECO, Aveiro Institute of Materials, Department of Chemistry, University of Aveiro, 3810-193 Aveiro, Portugal

**Keywords:** silver complex, H_2_TMPyP, metalloporphyrins, G-quadruplexes, telomerase, oncogene promoters, HaCaT cell line, singlet oxygen generation, PDT

## Abstract

Cancer is a leading cause of death, so continuous efforts into cancer therapy are imperative. In tumor cells, telomerase and oncogene activity are key points for uncontrolled cell growth. Targeting these processes with ligands that inhibit telomerase and/or reduce oncogene expression has been identified as a promising cancer therapy. This study evaluated the selectivity and affinity of the silver^II^ complex of 5,10,15,20-tetrakis(*N*-methyl-4-pyridinium)porphyrin (**AgTMPyP**) to stabilize DNA sequences capable of forming G4 structures mimicking the telomeric and oncogene regions, using spectroscopic, biochemical methods and in vitro assays. The tetracationic silver complex was compared with the free base, **H_2_TMPyP**, and the zinc^II^ complex, **ZnTMPyP**. The results obtained from UV-Vis and fluorescence methods pointed to a great affinity and good selectivity of **AgTMPyP** to G4 structures, especially for the oncogene *MYC.* In general, an increase in the ability of the studied ligands for ^1^O_2_ generation when interacting with oncogenic and telomeric G4 sequences was found. The results of the PCR stop assays proved that **AgTMPyP** has the ability to inhibit Taq polymerase. Additionally, in vitro assays demonstrated that the silver^II^ complex exhibits low cytotoxicity against HaCaT— an immortalized, non-tumorigenic, skin keratinocytes cell line—and, although nonexclusive, **AgTMPyP** shows nuclear co-localization.

## 1. Introduction

Chemotherapeutic drugs, commonly used in chemotherapy and radiotherapy, have a good success rate; however, their use is significantly limited by numerous side effects due to their lack of selectivity for tumor cells. Therefore, overcoming this disadvantage is of utmost importance for the development of new and alternative therapeutic strategies [1]. Among these alternative strategies are those that involve the study of telomeres and G-quadruplexes and their correlation with telomerase and oncogenes [2,3,4,5].

During cell division, interruptions in the DNA replication process are associated with the shortening of telomeres, located at the ends of chromosomes. However, the reverse transcriptase enzyme telomerase is able to protect telomeres from that shortening process by introducing repetitive and specific DNA sequences to the 3′ telomere end of chromosomes [6]. Nearly 90% of human malignancies have been shown to overexpress this enzyme [7], and telomerase has been identified as a target for the development of novel antitumor drugs.

The occurrence of tumors is closely associated not only with telomerase overexpression [6,8,9] but also with abnormal expression of oncogenes [10,11]. *MYC* is a proto-oncogene that initiates selective amplification of gene expression to promote processes such as cell growth and proliferation, among others [12,13]. When abnormally expressed, *MYC* gives rise to the oncogene *MYC*, which drives cancer development and metastasis and is present in up to 70% of human cancers. When oncogenes are produced, they stimulate cell multiplication and, thus, play a key role in cancer development [14]. For this reason, oncogenes have been the subject of extensive investigation as potential therapeutic targets for anticancer drugs [10,14,15].

Non-canonical secondary DNA structures formed in guanine-rich DNA sequences with sequential guanine repeats [16,17,18,19,20], known as G-quadruplexes (G4), can be found in functional regions of DNA, such as telomeres, oncogene promoters, ribosomal DNA, and the 5′ untranslated region, where critical processes such as replication, repair and transcription occur [3,16,21,22,23,24]. Genome sequencing has identified G4 structures in more than 700,000 distinct sites [25,26]. When stabilized by ligands, G4 structures can inhibit telomerase function and/or reduce oncogene expression [27].

The discovery that telomeres’ DNA sequences are guanine-rich telomeric repeats that could fold into G4 structures has motivated researchers to search for G4 stabilizing ligands that may restrict cancer cell growth by interfering with telomerase function [6,28,29]. Other studies have also noted that, when helicases are disturbed, G4 complexes can pose a roadblock to the replication fork movement; thus, treatment with molecules that possess the ability to stabilize G4 structures resulted in lower mRNA levels at genes containing G4 sequences in their promoters, such as the oncogenes *MYC* [11,30,31,32] and *KRAS* [21,33,34,35], confirming the theory that G4 structures are an obstacle to the transcription machinery’s development.

With the aim of interrupting the uncontrolled proliferation of cancer cells by inhibiting the function of the telomerase enzyme or reduc oncogene expression, and based on previous discoveries, new molecules with promising structural characteristics have been developed and tested for the stabilization of G-quadruplexes and the consequent inhibition of telomerase function or transcription downregulation. The binding of molecules that can promote the stabilization of these G4 structures could downregulate transcription events and/or block the telomeres in the G4 configuration, thus avoiding their elongation by telomerase, which only recognizes single-stranded DNA as sites of replication [2,36,37,38,39].

Numerous small ligands have been found to bind non-covalently to DNA and RNA G4 structures. These ligands predominantly consist of structures featuring fused or unfused aromatic macrocycles [32,40]. Porphyrins and phthalocyanines are two classes of ligands that, due to their structural characteristics and optical properties, have been widely studied and are recognized as classes of drugs with the potential to inhibit telomerase activity and/or compromise transcription events [21,41,42,43,44,45,46,47,48].

One of the most studied porphyrins in this field is the cationic 5,10,15,20-tetrakis(*N*-methyl-4-pyridinium)porphyrin (**H_2_TMPyP**). Although lacking the selectivity for G4 structures, this ligand has been reported for its high affinity and ability to stabilize not only telomeric G4 [49,50,51] but also biologically relevant non-telomeric G4 structures from human oncopromoters, bacterial genomes and viral genomes [52]. The corresponding zinc^II^ complex, **ZnTMPyP**, has also been widely studied and is noted to have good affinity and selectivity for G4 structures [53,54,55,56].

Silver complexes, along with their water solubility, stability, lipophilicity, and redox ability, are also described as showing greater cytotoxicity to cancer cells, mainly due to their stronger interactions with cellular targets such as DNA, proteins, and membranes, their enhanced ability to generate reactive oxygen species (ROS) and the potential release of cytotoxic silver ions. In contrast, zinc complexes, though biologically active, demonstrate lower toxicity because zinc is less reactive than silver [57]. Although the potential lethal effects of silver remain uncertain, in bacterial cells, its interaction with nucleic acids appears to be predominantly directed towards the DNA bases, rather than the phosphate groups [58]. The promising results published on silver complexes as antibacterial and antitumor agents [59,60], along with our preliminary results concerning the interaction of a series metalloporphyrins with different metal ions (Zn^II^, Ni^II^, Cu^II^, Ag^II^, Pd^II^, Mn^III^ and Co^III^) with the unimolecular telomeric sequence AG_3_(T_2_AG_3_)_3_, as well as shorter tetramolecular (TG_4_T)_4_ and bimolecular (G_4_T_4_G_4_)_2_ DNA sequences [41], prompted us to select the silver complex **AgTMPyP** for further evaluation.

In our previous work, we identified that the **AgTMPyP** complex exhibits excellent affinity and a predictable selectivity pattern toward all of the tetramolecular, bimolecular, and unimolecular sequences. So, our aim here was to evaluate whether **AgTMPyP** can selectively stabilize the telomeric G4 and the oncogene promotors *MYC* and *KRAS*. Compared to our previous publication [41], which focused on shorter tetramolecular, bimolecular, and unimolecular telomeric sequences, this study evaluates the later and G4 sequences present in oncogene promoters (*MYC* and *KRAS*). These longer sequences provide a more precise and biologically relevant model for studying the interactions of metalloporphyrins with DNA, enhancing the relevance and potential therapeutic applications of our findings. So, the selected sequences were chosen for two main reasons: (i) biological relevance, since the ability to form G4 structures with a higher number of base pairs (22 bp) allows for the formation of more complex and biologically relevant structures that better resemble those observed in living organisms, particularly in cancer cells; and (ii) therapeutic potential, since G4 structures in oncogene promoters, such as *MYC* and *KRAS*, play a critical role in gene regulation, making them important targets for therapeutic intervention.

For the selection of the double-stranded DNA oligonucleotide (ds26), we considered its comparable number of base pairs (26 bp) to the G4 sequences (22 bp). This similarity provides a meaningful comparison, in contrast to the previously selected salmon sperm DNA, which is a broader representation of DNA structure.

For comparative purposes, the free base **H_2_TMPyP** and the zinc^II^ complex **ZnTMPyP** were also studied. Since a high percentage of DNA exists in the double-stranded (dsDNA) form within the cellular environment, ligands should exhibit greater affinity and selectivity for G4 DNA structures. If not, ligands’ availability to bind G4 structures will be reduced due to their interactions with dsDNA. Thus, additionally, the dsDNA sequence (ds26) was also included to verify the affinity and selectivity patterns towards the G4 structures.

**Figure 1:**
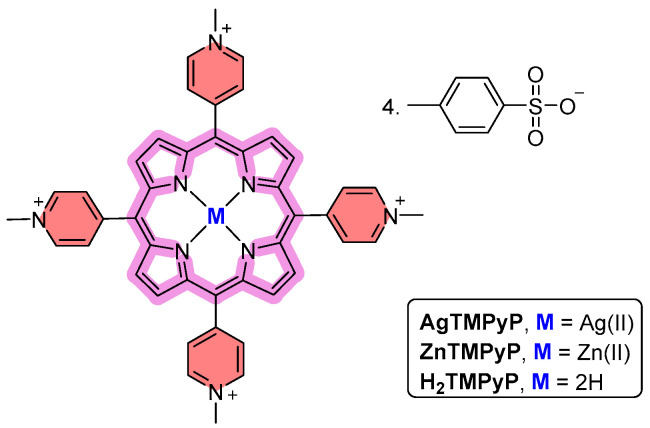
Chemical structure of cationic metalloporphyrins **AgTMPyP** and **ZnTMPyP,** and their free-base counterpart **H_2_TMPyP.**

Taking into account the optical properties of porphyrins, the interactions of **AgTMPyP**, **H_2_TMPyP,** and **ZnTMPyP** (Figure 1) with the selected G4 structures and the dsDNA sequence (ds26) were preliminarily evaluated using well-established biophysical and biochemical methodologies, namely, ultraviolet–visible (UV–Vis) spectroscopy and fluorescence spectroscopy. Considering the potential of a combined G4 stabilization and PDT approach as an antitumor strategy [61,62,63], the ability of the porphyrin–DNA adducts to generate ^1^O_2_ was also evaluated.

The results obtained with the **AgTMPyP** were further complemented using polymerase chain reaction (PCR) stop assays, cellular viability assays, and confocal microscopy, the latter using the immortalized HaCaT cell line, obtained from skin keratinocytes. This cell line is usually used in studies related to skin diseases and cancer, due to its spontaneous immortality, which is associated with mutations in the p53 tumor-suppressor gene [64,65,66]. These mutations reduce the cells’ ability to undergo apoptosis, thus contributing to uncontrolled proliferation. Conversely, an increased activity of the enzyme telomerase has been described as resulting in telomere length preservation [65,67,68,69]. So, despite these mutations, HaCaT cells retain normal differentiation, making them a versatile model for research on skin regeneration, toxicology, and cancer [64].

## 2. Results and Discussion

### 2.1. UV–Vis Spectroscopy

The ability of **AgTMPyP** to stabilize the telomeric G4 and the G4-forming sequences from the oncogene promotors *MYC* and *KRAS* (see Table S1 for the oligonucleotide sequences) was initially evaluated using Ultraviolet–Visible (UV–Vis) spectroscopy. For comparison, the double-stranded DNA sequence (ds26) was also included to verify the affinity and selectivity patterns. The behavior of the free base **H_2_TMPyP** and of the zinc^II^ complex **ZnTMPyP** was also analyzed under the same conditions in order to evaluate the impact of metal ions.

UV–Vis spectroscopy is a valuable spectroscopic technique frequently used to study ligands’ interactions with DNA structures, providing insights into ligands’ affinity, selectivity, and interaction modes [70,71].

This approach is particularly useful in the context of porphyrins, which exhibit a strong Soret band between 400 and 460 nm, accompanied by less intense Q bands between 500 and 650 nm; the presence of four Q bands is typically associated with the free-base form, while two bands are usually related to metalloporphyrins. By observing bathochromic or hypochromic shifts in these bands during titrations with DNA, particularly G4-forming sequences, one can infer different types of interactions between porphyrins and DNA [32,41,42,72,73].

The more significant changes in the UV-Vis absorption spectra are associated with a higher degree of contact between the π-systems of the interacting species. Groove or outside binding, involving less direct contact, typically results in red shifts (Δλ_max_) of less than 8 nm. In contrast, intercalation causes a notable decrease in intensity (over 35% hypochromism) and a simultaneous red shift of at least 15 nm [32,41,74].

**Figure 2:**
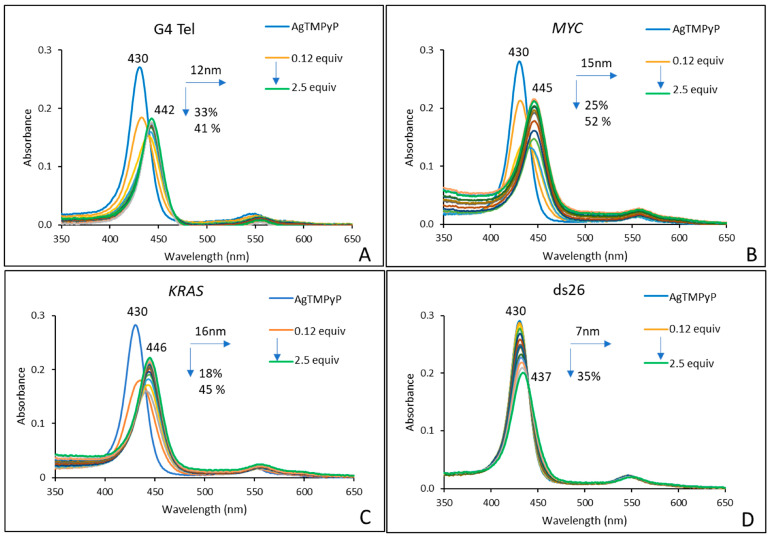
UV–Vis spectra obtained from the titration of **AgTMPyP** (2 × 10^−6^ M) in PBS (10 mM KH_2_PO_4_, 10 mM K_2_HPO_4_ and 100 mM KCl; pH 6.8), with 0.12 to 2.5 equiv. of (**A**) G4 Tel, (**B**) *MYC*, (**C**) *KRAS*, and (**D**) ds26.

**Figure 3:**
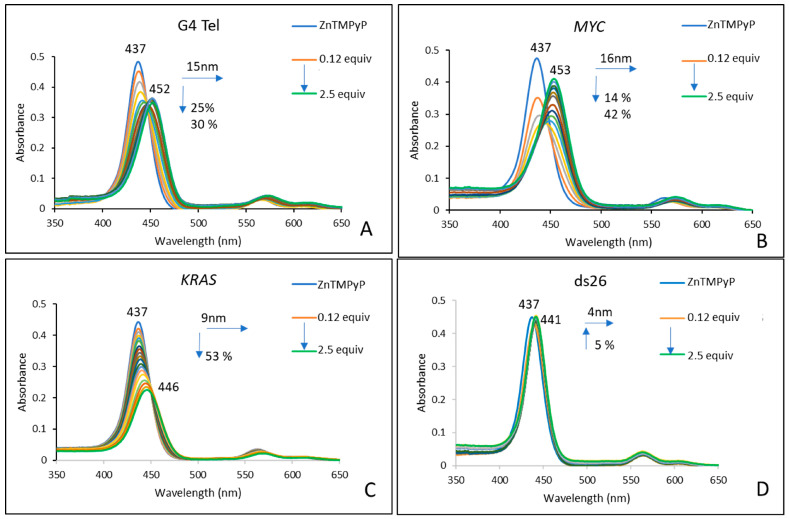
UV–Vis spectra obtained from the titration of **ZnTMPyP** (2 × 10^−6^ M) in PBS (10 mM KH_2_PO_4_, 10 mM K_2_HPO_4_, and 100 mM KCl; pH 6.8), with increasing amounts (0.125 to 2.5 equiv.) of (**A**) G4 Tel, (**B**) *MYC*, (**C**) *KRAS*, and (**D**) ds26.

Titrations were performed by gradually adding increasing amounts of the previously prepared DNA solutions to 2 × 10^−6^ M solutions of each porphyrin, both in PBS (10 mM KH_2_PO_4_, 10 mM K_2_HPO_4_, and 100 mM KCl; pH 6.8). All of the titrations were performed under identical experimental conditions for each ligand. The experiments were considered complete when, after three successive additions, no significant spectral changes were observed, corresponding to a total addition of 2.5 equivalents of each DNA sequence. The results obtained for **AgTMPyP** and each oligonucleotide sequence are presented in Figure 2, while those for **ZnTMPyP** and **H_2_TMPyP** are presented in Figure 3 and Figure S1, respectively.

The results from the titrations of **AgTMPyP** (Figure 2; Table 1, entries 1–4) show that both hypo- and bathochromic effects were observed at the end of all the titrations. The most pronounced modifications were observed for the oncogenes *KRAS* and *MYC*, which exhibited red shifts of 16 and 15 nm, respectively, accompanied by final hypochromism effects of 18 and 25%, respectively. For the telomeric G4 (G4 Tel), a red shift of 12 nm and hypochromism of 33% were observed.

The results for the double-stranded ds26 sequence showed slight changes, with a red shift of 7 nm and 35% hypochromism. These results are consistent with the data previously obtained with ds salmon DNA [41], appearing to indicate that **AgTMPyP** presents a better affinity for the sequences present in oncogenes.

The results for the **ZnTMPyP** complex (Figure 3; Table 1, entries 5–8) reveal a distinct behavior compared to that of the **AgTMPyP** complex. The most significant changes were observed with the G4 Tel and *MYC* sequences, with only small differences between the two. It is interesting to note that, apparently, the interaction between **ZnTMPyP** seems to be faster for the *MYC* sequence, since the first three additions caused a considerable red shift (7 nm) and hypochromism (57%). In contrast to the Ag^II^ complex **AgTMPyP**, the modifications of the spectra in the presence of *KRAS* were less pronounced, resulting in a red shift of 9 nm and hypochromism of 53%. The slight modifications observed with the double-stranded ds26, red shift of 4 nm and a hyperchromic effect of 5%, suggest a potential selectivity for G4 structures.

The results obtained for **H_2_TMPyP** (Figure S1, Table 1, entries 9–12) show significant spectral alterations in the presence of all DNA structures, with red shifts ranging from 13 to 19 nm and hypochromic effects between 31% and 65% at the end of the titrations. No major differences were found between the G4 structures, and the alterations, although slightly smaller for the ds sequence, were consistent with its lack of selectivity [42].

Concerning the affinity and selectivity of the studied porphyrins, the data obtained (Table 1 and Figure 1, Figure 2, and Figure S1) are coherent with those that we previously obtained for the same derivatives and other DNA sequences able to form G4 in other conformations, namely, tetramolecular and bimolecular intermolecular geometries [41]. It should be noted that the higher selectivity of **AgTMPyP** for G4 DNA over the double-stranded salmon sperm sequence, as found in our previous studies, is due to the use of heterogeneous salmon sperm DNA, which provided greater distinction with G4 structures. In the current study, using more uniform ds26 DNA with a similar number of base pairs reduced this difference, leading to lower selectivity.

Although the free-base porphyrin showed higher affinity for both of the selected oncogenic sequences, its lack of selectivity prevents the use of this promising ligand as G4-stabilizing agent; an efficient G4-stabilizing ligand should be capable of recognizing G4 DNA within the cellular nucleus, where a substantially higher amount of duplex DNA is present.

### 2.2. Fluorescence Spectroscopy

#### 2.2.1. Fluorescence Titrations

Based on the UV–Vis results, we decided to conduct fluorescence experiments to compare the ability of the selected metalloporphyrins, **AgTMPyP** and **ZnTMPyP**, to stabilize G4 structures present in telomeres (G4 Tel) and in oncogene sequences (*MYC* and *KRAS*). This analysis aimed to confirm selectivity for G4 structures and verify the existence of selectivity for specific G4 sequences.

The intrinsic fluorescence of porphyrins and their analogs is a significant advantage for studying ligand–DNA interactions, as it allows spectrofluorimetric titrations to complement the UV–Vis data. Fluorescence titrations often lead to quenching of the ligands’ fluorescence due to electron transfer between the excited ligand and the DNA structures [32,42]. This process is particularly favorable in G4 structures, which contain numerous guanines that are more easily oxidized than other DNA bases [75]. Additionally, since end-stacking or external binding with G-quartets is a common binding site for many ligands, including porphyrins, the binding of these fluorescent ligands typically results in effective fluorescence quenching.

**Figure 4:**
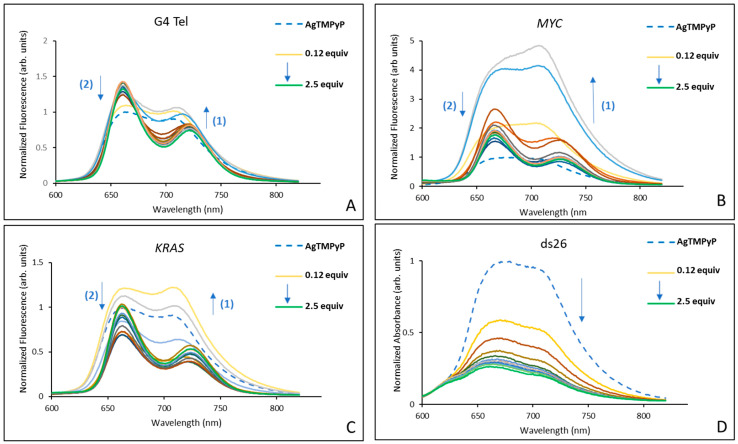
Fluorescence spectra obtained in the titrations of **AgTMPyP** (2 × 10^−6^ M) in PBS (10 mM KH_2_PO_4_, 10 mM K_2_HPO_4_, and 100 mM KCl; pH 6.8), with 0.125 to 2.5 equivalents of (**A**) G4 Tel, (**B**) *MYC*, (**C**) *KRAS*, and (**D**) ds26.

**Figure 5:**
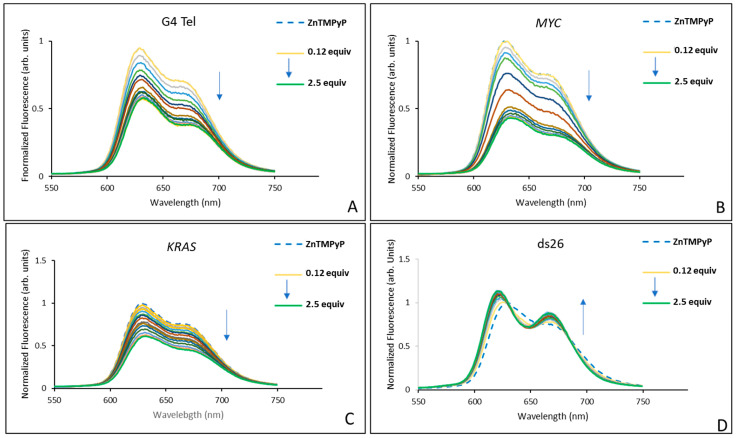
Fluorescence spectra obtained in the titrations of **ZnTMPyP** (2 × 10^−6^ M) in PBS (10 mM KH_2_PO_4_, 10 mM K_2_HPO_4_, and 100 mM KCl; pH 6.8), with 0.125 to 2.5 equivalents of (**A**) G4 Tel, (**B**) *MYC*, (**C**) *KRAS*, and (**D**) ds26.

The results obtained in the titrations of the complexes **AgTMPyP** and **ZnTMPyP** with each oligonucleotide sequence are presented in Figure 4 and Figure 5, respectively.

In accordance with the characteristic fluorescence spectra of porphyrins, the spectra of **AgTMPyP** showed a broad emission band with two nearly symmetric maxima regions at 664 nm and 706 nm. During the titrations, changes in the intensity and form of these bands were observed, and for sequences that are able to form G4 structures, the band at 664 nm is generally more pronounced at the end of titrations.

Interestingly, in the titrations with sequences that can form G4 structures, the porphyrin fluorescence increased during the first three additions, particularly with the *MYC* sequence, which showed about a 5-fold increase after the first DNA addition. Although the intensity decreased after the initial additions, the final intensity of the complex remained 1.8 times higher than that of the porphyrin alone. For the other two G4 sequences, the increase was less pronounced: 1.3-fold higher for G4 Tel, while the *KRAS* sequence had a similar final intensity to that of the initial porphyrin. In contrast, for the double-stranded sequence, there was no initial increase, and the fluorescence decreased by 75% throughout the titration. Although these results are consistent with the UV–Vis data (Table 1, entries 1 to 4), which showed that the silver complex had a binding affinity ranked as *KRAS* ≈ *MYC* > G4 Tel >> ds26, these data seem to suggest a stronger affinity of **AgTMPyP** for the *MYC* sequence.

The results summarized in Figure 5 for **ZnTMPyP** and for the same DNA sequences show a reduction in the fluorescence intensity for all of the G4 DNA sequences, but without significant alterations in the two bands observed for the initial complex: a more intense band at 630, and a less pronounced maximum at 670 nm.

This complex showed a similar quenching of 43% for G4 Tel and 39% for *KRAS*. A more pronounced quenching of 57% was observed for *MYC*. In the case of the double-stranded ds26, only a smaller enhancement of the fluorescence (about 13%) was observed. These results are consistent with those obtained from the UV–Vis data, suggesting a higher affinity of this ligand for the G4 structures.

The obtained data allowed us to calculate some of the apparent dissociation constants (K_D_), as well as the Hill coefficients, which are summarized in Table 2. However, the pattern of fluctuations observed in the fluorescence for **AgTMPyP** and both oncogenes prevented the determination of their K_D_ values, giving rise to unacceptable error margins and potentially inconsistent conclusions.

The biphasic fluorescence response, characterized by an initial fluorescence enhancement after the first two DNA additions, followed by a decrease with further additions, made it impossible to fit the data to a binding model in the case of **AgTMPyP** with *MYC* and *KRAS*. The initial fluorescence enhancement likely arose from high-affinity interactions between the ligand and specific binding sites on the G4 structure, such as grooves, loops, or terminal G-tetrads, which stabilize the ligand within a structured environment. As more G4 DNA was added, these high-affinity sites became saturated, leading to subsequent binding at lower-affinity sites or through less favorable modes, such as electrostatic interactions with the DNA backbone or nonspecific aggregation; these lower-affinity interactions can contribute to fluorescence quenching.

The fluorescent intercalator displacement (FID) assay was performed to complement these studies, and the results are presented below in Section 2.2.2.

The Hill coefficient (n) is a parameter that reflects the cooperativity of ligand binding to DNA structures, indicating how the binding of one ligand influences the binding of others. Hill coefficients higher than 1 are indicative of positive cooperativity, where the binding of one ligand increases the probability of additional ligand binding. Values lower than 1 are indicative of negative cooperativity, meaning that the binding of one ligand decreases the probability of additional ligand binding. As summarized in Table 2, in the present study, negative cooperativity was found for both complexes with the double-chain sequence ds26, suggesting that the first binding event reduces the affinity for subsequent ligands, possibly due to steric hindrance or conformational changes in the DNA or ligand [76]. For **AgTMPyP** with G4 Tel (n = 1), the data suggest that each ligand binds independently of the others, which is also consistent with non-cooperative binding. In contrast, the **ZnTMPyP** values, ranging from 1.35 to 3.85, indicate positive cooperativity. This suggests that the initial binding of **ZnTMPyP** stabilizes key structural features of the G4 Tel DNA, such as the grooves, loops, or G-tetrads, creating a more favorable environment for subsequent ligands to bind. Higher Hill coefficients, such as 3.85, may reflect multiple high-affinity binding sites that cooperate to enhance ligand affinity as the DNA–ligand complex stabilizes. This stabilization could also involve changes in the conformation of the DNA, further optimizing the binding interactions.

#### 2.2.2. Fluorescence Intercalator Displacement (FID) Studies

The fluorescent intercalator displacement (FID) assay is a fluorescence-based method that is commonly used to evaluate and confirm the affinity and selectivity of ligands for G4 structures [70].

**Figure 6:**
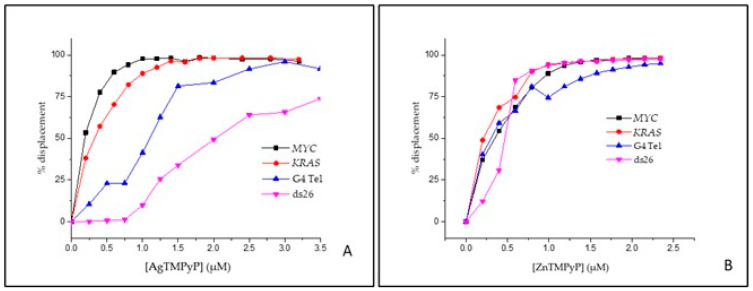
Results from FID experiments for (**A**) **AgTMPyP** and (**B**) **ZnTMPyP**. TO (3.5 µM)–DNA (1 µM) adduct solutions prepared in PBS (10 mM KH_2_PO_4_, 10 mM K_2_HPO_4_, and 100 mM KCl; pH 6.8).

This methodology is based on the displacement of a fluorescent probe, such as thiazole orange (TO), by a ligand from the TO adduct with DNA in PBS. When a ligand is added to the DNA (1 µM)–TO (3.5 µM) adduct, it competes with TO for binding, causing a decrease in fluorescence [77,78]. The affinity and selectivity of a ligand for the different DNA structures can be assessed based on the ligand concentration required to reduce TO fluorescence by 50%, a value identified as DC_50_. Additionally, these values allow for the calculation of the G4 selectivity factor ^G4^S (^G4^S = ^ds^DC_50_/^G4^DC_50_) of each ligand for G4 DNA versus double-stranded DNA; a ^G4^S value superior to 1 indicates selectivity for G4, while ^G4^S ≤ 1 indicates non-selectivity [79]. To clarify the aforementioned data, the titrations of each DNA–TO adduct were performed in the presence of the complexes **AgTMPyP** and **ZnTMPyP,** and the results obtained are shown in Figure 6 and compiled in Table 2. As an example, the series of spectra obtained by titrating TO–DNA adducts with the zinc complex are presented in Figure S2.

It is evident that both complexes show selectivity for the G4 structures, since the DC_50_ values obtained are lower than those obtained for ds26, leading to ^G4^S values higher than 1 (Table 2). We can also conclude that the **AgTMPyP** complex has a much higher selectivity for the oncogene sequences *MYC* and *KRAS* compared to G4 Tel, with ^G4^S values of 11.1, 7.1, and 1.8, respectively. These results corroborate the previous data from the UV–Vis experiments, which pointed to a higher affinity of this complex for oncogenes. Although the selectivity of **ZnTMPyP** for G4 structures is evident, the difference in selectivity towards any G4 structure is less pronounced, with ^G4^S values of 3.25 for *MYC*, 1.91 for G4 Tel, and 1.75 for *KRAS*.

#### 2.2.3. Singlet Oxygen Generation by Porphyrin–DNA Conjugates

**Figure 7:**
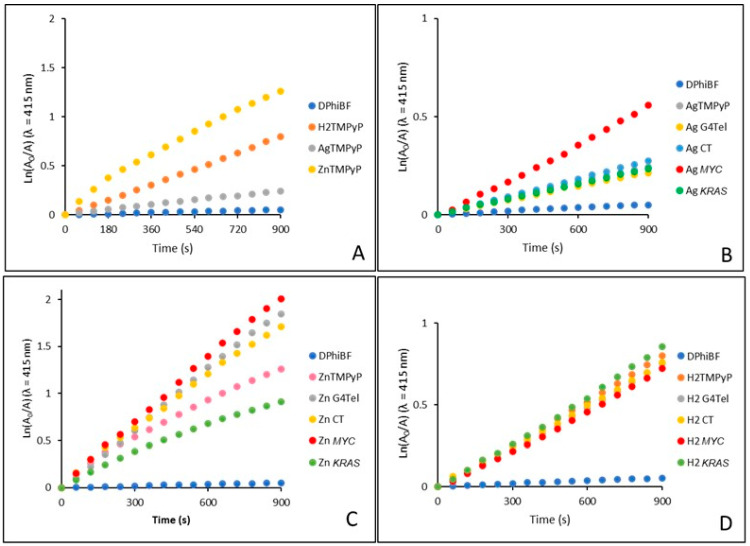
Time-dependent photodecomposition of DPiBF at 50 × 10^−6^ M in the presence of (**A**) **AgTMPyP, ZnTMPyP,** and **H_2_TMPyP** (0.5 × 10^−6^ M); (**B**) comparison between **AgTMPyP** and **AgTMPyP**–DNA adducts; (**C**) comparison between **ZnTMPyP** and **ZnTMPyP**–DNA adducts; (**D**) comparison between **H_2_TMPyP** and **H_2_TMPyP**–DNA adducts.

The well-documented ability of porphyrins to produce cytotoxic reactive oxygen species (ROS)—namely, singlet oxygen (^1^O_2_), which can lead to cellular death—is responsible for their success as photosensitizers (PSs) in photodynamic therapy (PDT). Generally, it is well accepted that porphyrin-based PSs induce cell death via ^1^O_2_ generation. Considering the potential of combining G4 stabilization with PDT as an antitumor strategy, we decided to evaluate whether the conjugates formed in the presence of DNA sequences retain the efficiency of porphyrins in producing ^1^O_2_ before conjugation. These experiments were performed by an indirect method that measures the absorption decay of the ^1^O_2_ scavenger diphenylisobenzofuran (DPiBF) at 415 nm with red-light irradiation (630 ± 20 nm) and an irradiance of 11.0 mW.cm^−2^. The ^1^O_2_ generated by the PS will be involved in a [4 + 2] cycloaddition reaction with the DPiBF present in the solution, with formation of the colorless dibenzoylbenzene [80,81,82]. The photodecay rates of DPiBF in the absence and in the presence of the selected complexes **AgTMPyP**, **ZnTMPyP,** and the free-base porphyrin **H_2_TMPyP** are presented in Figure 7.

As can be seen in Figure 7A, upon irradiation, all of the porphyrin-based PSs were able to induce a reduction in DPiBF, with the zinc^II^ complex **ZnTMPyP** presenting a remarkable ability to promote photodecomposition of DPiBF. This ability was considerably higher than that observed for the other porphyrins, especially when compared with **AgTMPyP** (≈1.5-fold lower). It is interesting that the presence of certain DNA structures, particularly in the case of the complexes, can lead to a significant improvement in their ability to promote DPiBF’s photodecomposition (Figure 7B,C). For both complexes, the highest increase was observed after the interaction with the DNA structure *MYC* (30% for **AgTMPyP** and 50% for **ZnTMPyP**), when compared with the free ligand. For **H_2_TMPyP**, no noticeable alterations were observed (Figure 7C).

The remarkable ability of the zinc^II^ complex in the presence of *MYC* and G4Tel must be highlighted, as its efficiency in the absence of DNA structures is already approximately 30% higher than the value observed for **H_2_TMPyP**. This porphyrin is well known as an effective ^1^O_2_ generator and is usually selected as a reference in PDT and antimicrobial photodynamic therapy (aPDT) experiments [83,84]. A similar behavior was recently reported by us in a study involving porphyrin–triphenylphosphonium derivatives and G4 stabilization [61].

The results demonstrated the high capacity of porphyrins to generate ROS, specifically ^1^O_2_, even during their interaction with G4 structures. The obtained results suggest that the less-studied **AgTMPyP** could be employed as a dual-target agent, stabilizing DNA G4 structures, regulating the *MYC* expression in the *MYC* gene promoter, and inducing cell death through photodynamic processes.

### 2.3. Polymerase Chain Reaction (PCR) Stop Assay

The PCR stop assay is a biochemical method that is commonly used to study ligands’ affinity to oligonucleotides, i.e., sequences that are able to form G4 structures. Its frequent application in G4 studies is well documented in the literature, as highlighted by Jaumot and Gargallo [85]. In this assay, oligonucleotides are used as templates for primer extension, which is performed with a PCR primer, with a sequence that presents complementary to the terminal G-repeat. Following the extension conducted by the polymerase, a PCR product is obtained and amplified under specific PCR conditions [86].

The principle of the technique assumes that, in the presence of a G4-stabilizing ligand, the DNA polymerase is incapable of traversing these structures; it slows or stops since it is unable to efficiently resolve the quadruplex DNA. This means that the primer annealing and downstream extension are both inhibited, resulting in the formation of a lower amount of PCR products [87]. This assay can thus be used to evaluate the interaction of ligands with the G4 structures, since the stabilization of the oligonucleotide sequence by ligands will trigger a pause in Taq polymerase activity [49,70].

**Figure 8:**
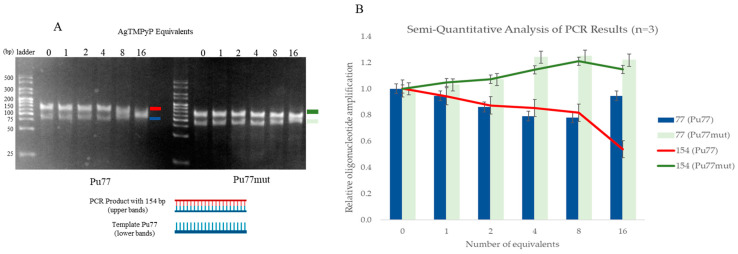
(**A**) Polyacrylamide gel electrophoresis of Pu77 (left) and Pu77-mut (right) PCR amplification products in the presence of increased amounts (0 to 16 equivalents) of **AgTMPyP** (1 equivalent = 4 × 10^−7^ M). (**B**) Representative analysis of the PCR products. The same colors code is represented in both figures.

To perform the PCR stop assay, we incubated the sequences of the Pu77 G4 (*MYC*) in the presence of the corresponding complementary sequence (Pu28) (Table S1) and of increasing concentrations of each ligand, involving 30 PCR cycles. The results are presented in Figure 8.

Figure 8 shows two bands of DNA per lane. According to the ladder, the lower band, at approximately 75 base pairs (bp), corresponds to the Pu77 template (which has 77 bp), while the upper band corresponds to the PCR product formed, that corresponds to the duplication of the Pu77 template with approximately 154 bp.

Furthermore, in the presence of increasing amounts of **AgTMPyP**, a significant decrease in the intensity of the PCR product band (upper band) is observed. The PCR product formation decreases by nearly 50% in the lane corresponding to 16 eq, which is accompanied by an increase in the intensity of the template band (Pu77). This behavior is likely related to the inability of Taq polymerase to complete its function, and lesser amounts of PCR products are formed. In turn, the lowered Taq polymerase activity can be directly related with the presence of the ligand and its contribution to G4 stabilization, by comparing the reaction between the ligand and the mutated sequence Pu77-mut used as a negative control. During the PCR with this sequence, even in the presence of the ligand, no inhibition is expected, since the sequence is not able to form G4 structures and, therefore, our ligand cannot interfere in the inhibition of the Taq polymerase’s action. Hence, the PCR product formation occurs normally in the presence of the mutated sequence.

Using the Image Lab software 6.1, we were able to take a mathematical approach to the interpretation of our results and translate them to a representative analysis and further comparison (Figure 8B).

The obtained graphic supports the previous gel interpretation. By analyzing the graphic bars, it is possible to confirm that the PCR product of the mutated Pu77, as well as the template, suffered no changes regarding any type of inhibition; in fact, it is possible to observe a slight increase in their percentages up until the 16 equivalents, where the concentration of the ligand is high enough to prevent the occurrence of any interference.

Concerning the Pu77 template, the inhibition of the Taq polymerase is confirmed by the decrease in the percentage of PCR products and the consequent increase in the free Pu77 template, which is no longer used for the polymerase chain reaction.

Taken together, these results confirm the ability of the Ag^II^ metalloporphyrin to inhibit Taq polymerase. The final PCR products are suppressed in a dose-dependent manner by ligand stabilization of the *MYC* G4 structure.

### 2.4. Cell Viability and Confocal Microscopy

#### 2.4.1. Cell Viability

To assess the in vitro activity of the studied compounds, it is essential to accurately predict the potential adverse effects of the selected compounds and to detect any toxicity levels. Alamar Blue is a redox indicator that is commonly employed to assess the metabolic activity and cellular health, and it is also used to ascertain cell viability and cytotoxicity in a range of biological systems and in diverse cell types [88,89].

**Figure 9:**
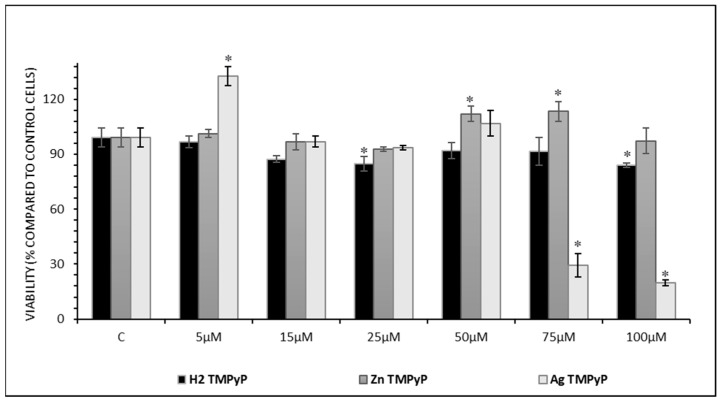
Viability profile of HaCaT cell culture upon 24 h of incubation with 5 to 100 × 10^−6^ M of **AgTMPyP**, **ZnTMPyP,** and **H_2_TMPyP**. Results are expressed as the mean ± SD, where ∗ indicates statistically significant differences compared to the control with *p* < 0.05.

Thus, preliminary experiments were performed using the two complexes and the free base at concentrations ranging between 5 × 10^−6^ and 100 × 10^−6^ M during 24 h of cellular incubation, and the cellular viability was determined through the Alamar Blue assay [90]. Figure 9 shows the viability data profile for the selected ligands in the chosen range of concentrations. In the case of the silver complex, concentrations equal and superior to 75 × 10^−6^ M proved to be cytotoxic for HaCaT cells, while concentrations of and below 50 × 10^−6^ M were well tolerated. This behavior seems to corroborate previous studies, where silver complexes were described as showing greater cytotoxicity to cancer cells than zinc complexes as mentioned above [57]. Interestingly, at 5 µM **AgTMPyP**, cell viability was significantly increased compared to the control. Similarly, **ZnTMPyP** showed a significant increase in cell viability at 50 and 75 µM. These significant increases in the cell viability suggest that, at these concentrations, the complexes are not harmful and may even support normal cell functions.

For the free-base porphyrin and its zinc complex, almost no viability loss was observed in the studied range of stimuli concentrations. For this reason, the chosen concentration for the co-localization experiments was 50 × 10^−6^ M for the studied metalloporphyrins. Each assay was run in triplicate, with the standard deviations being represented as error bars, and growth medium was used to incubate control samples.

As summarized in Figure 9, only the silver complex (**AgTMPyP**) significantly affected cell viability, with a reduction of approximately 75% at 75 × 10^−6^ M. This substantial decrease highlights its potential to compromise cellular functions and inhibit cell growth.

#### 2.4.2. Confocal Microscopy

**Figure 10:**
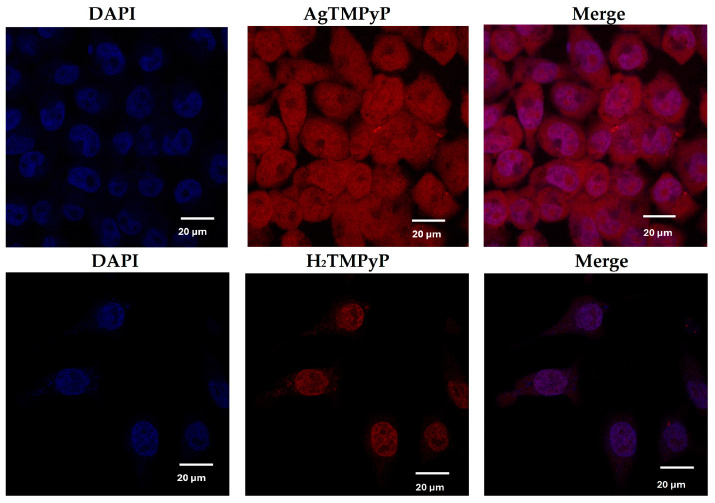
Confocal images of HaCaT cells treated for 24 h with **AgTMPyP** and **H_2_TMPyP**, both at a concentration of 50 × 10^−6^ M. DAPI was used as a reference for nucleus co-localization.

Confocal fluorescence microscopy was used to determine the localization of **AgTMPyP** in HaCaT cells, since these ligands have intrinsic fluorescence properties. Incubation of **AgTMPyP** at a concentration of 50 × 10^−6^ M for 24 h demonstrated that the silver complex was effectively internalized by HaCaT cells, with its distribution observed throughout the cell (Figure 10). Not only was it localized in the nucleus, it was also present in the cytoplasm. This distribution may indicate that **AgTMPyP** interacts with multiple cellular compartments, which could be relevant for its potential cytotoxic effects and activity in G4 stabilization. The **H_2_TMPyP** ligand, used as the reference, seemed to be less internalized, showing itself to be essentially accumulated in the cell nucleus.

The intrinsic fluorescence of **AgTMPyP,** in association with its co-localization in the cell nucleus, emphasizes its notable properties and potential for application in G4 labeling.

## 3. Materials and Methods

### 3.1. Chemicals

The chemicals were purchased as analytical grade and used without further purification. 5,10,15,20-Tetrakis(1-methylpyridinium-4-yl)porphyrin tosylate (**H_2_TMPyP**) was purchased from Sigma Aldrich (Aldrich, Steinheim, Germany), and the Ag^II^ and Zn^II^ porphyrin complexes were synthesized and characterized as previously described [41,61,91]. The molar extinction coefficients provided in datasheets or previously described, in phosphate-buffered. saline (PBS), were used for DNA sequences and for **H_2_TMPyP** (ε = 226,000, λ_max_ = 421 nm), **AgTMPyP** (ε = 136,014, λ_max_ = 430 nm), and **ZnTMPyP** (ε = 225,000, λ_max_ = 437 nm). Stock solutions of each porphyrin (1 × 10^−3^ M) were prepared in DMSO and stored at 4 °C. The required concentration of each porphyrin (2 × 10^−6^ M) was obtained by dilution of an aliquot of the stock solution in PBS.

### 3.2. Preparation of DNA Structures

The lyophilized DNA oligonucleotides were purchased from Eurogentec (Seraing, Belgium), and DNA stock solutions were prepared in Milli-Q water and diluted in phosphate-buffered saline (PBS) when required.

A solution containing 20 mM phosphate buffer (10 mL of KH_2_PO_4_ 1 M and 200 μL of K_2_HPO_4_ 1 M) and 100 mM KCl, with a pH 6.8, was used as the solvent for oligonucleotide solutions.

To ensure proper folding into quadruplex or ds structures, each oligonucleotide was heated to 85 °C for 10 min after solubilization in PBS and then allowed to cool overnight. The solutions were kept in storage at −20 °C. The sequences and abbreviations of the studied ds and G4 DNA structures are presented in Table S1.

### 3.3. UV–Vis Spectroscopy

The UV–-Vis titrations were performed by adding increasing amounts of each oligonucleotide solution (ranging from 0 to 2.5 equivalents) to each porphyrin solution in PBS at 2 × 10^−6^ M. The titrations were considered complete when no meaningful absorbance changes were observed after three successive additions [92]. Blank assays were carried out by adding small amounts of PBS buffer to the ligand solution. To confirm the results’ reproducibility, the experiments were performed in triplicate.

All of the UV–Vis absorption spectra were recorded on a Shimadzu UV-2501-PC spectrophotometer (Shimadzu Corporation, Kyoto, Japan), equipped with a Huber Compatible Control CC1, in the range of 350–750 nm, using a 1 cm length quartz cuvette.

For experimental details, please see the Supplementary Materials.

### 3.4. Fluorescence Spectroscopy

#### 3.4.1. Fluorescence Titrations

Fluorescence titrations were performed by adding increasing amounts of each oligonucleotide solution (ranging from 0 to 2.5 equivalents) to each porphyrin solution in PBS, at a concentration of 2 × 10^−6^ M, in a 1 × 0.4 cm semi-micro cuvette. Fluorescence emissions were collected in the range of 550–850 nm by exciting ligands at the wavelength corresponding to their UV–Vis spectrum maximum after each titration addition, using a Horiba FluoroMax-4 spectrofluorometer (Horiba, Kyoto, Japan). Experimental emissions were corrected using the following equation:

(1) $$I_{corr}=I_{obs}\times 10^{-\frac{A_{ex}\times d_{ex}}{2}-\frac{A_{em}\times d_{em}}{2}}$$

where d*_ex_* and d*_em_* are the path lengths corresponding to 1 cm and 0.4 cm, respectively [93,94]. These experiments enabled us to assess the apparent dissociation constants (*K*_D_) between each ligand and DNA structure. For experimental details, please see the Supplementary Materials.

#### 3.4.2. Fluorescence Intercalator Displacement (FID) Studies

Stock solutions of 35 × 10^−6^ M for thiazole orange (TO) and 10 × 10^−6^ M for the different oligonucleotides were prepared. The solutions were then gently mixed for 10 min on an orbital shaker, in order to promote the formation of 1:1 TO (3.5 µM)–DNA (1.0 µM) adducts. The fluorescence of the obtained adducts was confirmed using a FluoroMax-4 spectrofluorometer ((Horiba, Kyoto, Japan)), with an excitation wavelength of 485 nm and emission acquisition fixed between 510 and 750 nm, with slit widths set at 5 nm.

Each ligand was then added to the TO–oligonucleotide solution at concentrations ranging from 0 to 4 × 10^−6^ M. The resulting fluorescence was measured and led to the determination of the percentage of displacement, allowing us to further obtain the ligand concentration (DC_50_) required to displace 50% of the TO probe from the DNA structure. Once the DC_50_ for both the G4 (^G4^DC_50_) and ds (^ds^DC_50_) sequences was obtained, the selectivity factor to G4 structures (^G4^S) could also be defined [79]. For experimental details please, see the Supplementary Materials.

### 3.5. Singlet Oxygen Generation by Porphyrin–DNA Conjugates

For comparison, the efficiency of **AgTMPyP**, **ZnTMPyP,** and **H_2_TMPyP** to generate singlet oxygen (^1^O_2_) was first evaluated using solutions of 1,3-diphenylisobenzofuran (DPiBF) at a concentration of 50 × 10^−6^ M, and of each porphyrin derivative at 0.5 × 10^−6^ M, in dimethylformamide (DMF). Each solution, in a glass cuvette (1 × 1 cm), was then irradiated with a red-light LED board (630 ± 20 nm) at an irradiance of 11 mW.cm^−2^ for 900 s, under magnetic stirring.

To assess the ^1^O_2_ generation ability of each porphyrin–DNA conjugate, the hybrids were previously prepared by mixing each porphyrin (0.5 × 10^−6^ M) with one equivalent of the adequate DNA structures able to form G4 or ds structures. After adduct formation, the adducts were also irradiated under the same conditions described for the free PSs, in the presence of DPiBF (50 × 10^−6^ M). Results for control assays using only the solution of DPiBF (50 × 10^−6^ M) in DMF were also obtained.

### 3.6. PCR Stop Assay

The DNA sequences used in these experiments, Pu77, Pu77-mut, and the primer Pu28, are presented in Table S1. Oligonucleotides (10 × 10^−6^ M) were annealed in 20 mM PBS by heating to 85 °C for 10 min, and then slowly cooled to room temperature. Each reaction mixture was performed to a final volume of 25 µL containing the annealing buffer, 0.2 × 10^−3^ M dNTPS, 4 × 10^−7^ M of each DNA template, and 1 × 10^−7^ M primer. The reactions were further incubated with adequate concentrations of the ligands (0.4 to 6.4 × 10^−6^ M) for 1 h at room temperature. Thereafter, 1 unit of *Taq* DNA polymerase was added, and the reaction mixture was incubated in a thermal cycler (Bio-Rad, Hercules, CA, USA) under the following PCR conditions: 2 min at 94 °C, 30 cycles at 94 °C for 30 s, 58 °C for 30 s, and 72 °C for 30 s. The PCR products were then resolved on a 15% non-denaturing polyacrylamide gel and stained with stains-all solution. Experimental details are presented in the Supplementary Materials.

### 3.7. Cell Viability Assay

HaCaT cells were maintained in culture at 37 °C and 5% CO_2_ in an incubator and sub-cultured every three days through trypsinization using a 0.05% (*m*/*v*) ethylenediaminetetraacetic acid (EDTA) solution and trypsin/EDTA 0.05% (*m*/*v*) in Hanks’s Balanced Salt Solution (HBSS).

The Alamar Blue assay was used to determine cell viability in the presence of each porphyrin [90]. Dose–response experiments were performed for time periods of 24 h, by exposing cells to a range of porphyrin concentrations, in order to determine the EC_25_. Cells were kept in fresh culture medium (ctrl) or treated with the porphyrin complexes **AgTMPyP** and **ZnTMPyP** or the free-base derivative **H_2_TMPyP** in the range of concentrations from 5 × 10^−6^ to 100 × 10^−6^ M. The assays were performed in triplicate. For experimental details, please see the Supplementary Materials.

### 3.8. Confocal Microscopy

HaCaT cells were grown on glass coverslips in 12-well plates at a density of 3.5 × 10^4^ cells/mL and then incubated for 24 h at 37 °C and 5% CO_2_ for cell attachment. Subsequently, the cells were exposed to 50 × 10^−6^ M of each porphyrin for 24 h. After being washed 3 times with warm PBS, the cells were fixed with 4% paraformaldehyde in PBS for 15 min at room temperature and mounted onto glass slides with 4′,6-diamidino-2-phenylindole (DAPI)-containing Vectashield mounting medium (Vector Labs, Miraflores, Portugal). Microphotographs were acquired on a Zeiss LSM 880LSM 510 META confocal microscope (Zeiss, Jena, Germany). Further details are presented in the Supplementary Materials.

### 3.9. Statistical Analysis

The results of cell viability are expressed as the mean ± standard deviation (SD) of at least three replicates, being considered statistical significantly different at *p* < 0.05. The statistical analysis of the data was performed by one-way ANOVA, followed by Dunnett’s test, using Sigma Plot 14.0 software (Systat Software Inc., Palo Alto, CA).

## 4. Conclusions

The systematic analysis performed using spectroscopic techniques to compare the ability of **AgTMPyP**, **ZnTMPyP**, and the free base **H_2_TMPyP** to stabilize biologically relevant G4 DNA structures—particularly those associated with cancer-related sequences such as G4 Tel, *MYC*, and *KRAS*, revealed that **AgTMPyP** is a promising new ligand for stabilizing G4 structures, especially oncogenic sequences, followed by **ZnTMPyP**. The free base **H_2_TMPyP** showed interactions with both G4 structures and the double-stranded DNA (ds26), limiting its effectiveness as a G4 stabilizer in biological systems, where duplex DNA is predominant.

Both **AgTMPyP** and **ZnTMPyP** demonstrated selectivity for G4 structures, with DC_50_ values lower than those obtained for the double-stranded DNA (ds26). However, **AgTMPyP** displayed higher selectivity for the oncogene sequences *MYC* and *KRAS* compared to G4 Tel, with ^G4^S values of 11.11, 7.14, and 1.78, respectively. In contrast, **ZnTMPyP** showed less pronounced differences in selectivity, with ^G4^S values of 3.25 for *MYC*, 1.91 for G4 Tel, and 1.75 for *KRAS*. These differences may be attributed to structural changes induced by the metal ions, which result in more or less distorted porphyrin structures, affecting their interactions with the DNA sequences. Additionally, the ability of Zn^II^ to form additional interactions with nitrogen-rich DNA molecules may explain the smaller differences in **ZnTMPyP** selectivity among the G4 structures.

The obtained results also allowed us to conclude that the ability of **AgTMPyP**, **ZnTMPyP**, and **H_2_TMPyP** to generate singlet oxygen (^1^O_2_), a key reactive oxygen species (ROS) for photodynamic therapy (PDT), remains unaffected or even improved after their interaction with telomeric and oncogenic G4 structures. The order of their singlet oxygen generation efficiency in solution, —**ZnTMPyP > H_2_TMPyP > AgTMPyP**, is correlated with their intersystem crossing (ISC) efficiency, reflecting the influence of their metal centers, the planarity of the porphyrin macrocycle, and potential aggregation effects. Zn^II^, being diamagnetic, results in a higher ISC efficiency than the free base and the paramagnetic silver porphyrin. So, the enhanced ability of both **AgTMPyP** and **ZnTMPyP** to generate ^1^O_2_ while interacting with telomeric and oncogenic G4 structures, particularly the *MYC* sequence, is likely related to their reduced tendency to aggregate in the presence of this DNA sequence.

PCR stop assays using the Pu77 (*MYC*) sequence in the presence of its complementary strand (Pu28) revealed that **AgTMPyP** could suppress the formation of the double-stranded PCR product (Pu154) when present at 16 equivalents. This inhibition was likely due to stabilization of the *MYC* G4 structure, although further studies are required to fully understand the mechanism of action. These findings demonstrated its ability to inhibit Taq polymerase, suppressing PCR product formation in a dose-dependent manner.

Interestingly, only **AgTMPyP** significantly reduced the viability of immortalized HaCaT cells at 75 × 10^−6^ M within the evaluated concentration range (5 to 100 × 10^−6^ M), highlighting its potential to disrupt cellular functions and inhibit cell growth. Furthermore, the co-localization of **AgTMPyP** in the nuclei of HaCaT cells at concentrations below 75 × 10^−6^ M, although not exclusive, along with its intrinsic fluorescence, emphasizes its potential for G4 labeling applications and cellular imaging.

Altogether, these results suggest that **AgTMPyP** might have the potential to be used in a dual therapeutic approach. The obtained findings seem to suggest that a combination of G4 DNA stabilization and photodynamic therapy might be effective in targeting cancer cell proliferation by downregulating oncogene transcription and photoinducing cytotoxicity in cancer cells.

## Figures and Tables

**Table 1:** UV–Vis data from titrations of **AgTMPyP**, **ZnTMPyP,** and **H_2_TMyP** with selected DNA structures.

Entry	Oligonucleotides	λi(nm)	λf(nm)	Δλ (nm)	% Hypochromic Effect * Max/Final
	Porphyrin			**AgTMPyP**	
(1)	G4 Tel	430	442	12	41/33
(2)	*MYC*		445	15	52/25
(3)	*KRAS*		446	16	45/18
(4)	ds26		437	7	35
	Porphyrin			**ZnTMPyP**	
(5)	G4 Tel	437	452	15	30/25
(6)	*MYC*		453	16	42/14
(7)	*KRAS*		446	9	53
(8)	ds26		441	4	5/+5
	Porphyrin			**H_2_TMPyP**	
(9)	G4 Tel	421	435	14	40/35
(10)	*MYC*		439	18	53/34
(11)	*KRAS*		440	19	65
(12)	ds26		434	13	37/31

*—% hypochromism maxima and at the end of titration.

**Table 2:** Apparent equilibrium dissociation constants (K_D_) obtained from fluorescence titrations. DC_50_ values and the selectivity factor (^G4^S) obtained from G4-FID experiments.

**AgTMPyP**				
	G4 Tel	*MYC*	*KRAS*	ds26
K_D_/µM	0.63 ± 0.29	n.a.	n.a.	0.01 ± 0.007
n	1.0	---	---	0.49
DC_50_/µM	1.12 ± 0.27	0.18 ± 0.03	0.28 ± 0.01	2.00 ± 0.37
^G4^S	1.8	11.1	7.1	1.0
**ZnTMPyP**				
	G4 Tel	*MYC*	*KRAS*	ds26
K_D_/µM	1.30 ± 0.02	1.32 ± 0.13	2.72 ± 0.42	2.69 ± 0.02
n	1.35	3.85	1.59	0.67
DC_50_/µM	0.34 ± 0.02	0.20 ± 0.02	0.37 ± 0.01	0.65 ± 0.04
^G4^S	1.9	3.3	1.8	1.0

n.a.—not available; n—Hill coefficients.

## Data Availability

Data will be made available upon request.

## Supplementary Materials

The following supporting information can be downloaded at https://www.mdpi.com/article/10.3390/ijms252413556/s1.

## References

[1] Mbugua S.N., Njenga L.W., Odhiambo R.A., Wandiga S.O., Onani M.O. (2021). Beyond DNA-targeting in Cancer Chemotherapy. Emerging Frontiers—A Review. Curr. Top. Med. Chem..

[2] Sekaran V., Soares J., Jarstfer M.B. (2014). Telomere maintenance as a target for drug discovery. J. Med. Chem..

[3] Xu Y., Goldkorn A. (2016). Telomere and telomerase therapeutics in cancer. Genes.

[4] Balagurumoorthy P., Brahmachari S.K. (1994). Structure and stability of human telomeric sequence. J. Biol. Chem..

[5] Cech T.R. (2004). Beginning to understand the end of the chromosome. Cell.

[6] Nandakumar J., Cech T.R. (2013). Finding the end: Recruitment of telomerase to telomeres. Nat. Rev. Mol. Cell Biol..

[7] Chen Y., Zhang Y. (2016). Functional and mechanistic analysis of telomerase: An antitumor drug target. Pharmacol. Ther..

[8] Bailey S.M., Murnane J.P. (2006). Telomeres, chromosome instability and cancer. Nucleic Acids Res..

[9] Shay J.W., Bacchetti S. (1997). A survey of telomerase activity in human cancer. Eur. J. Cancer.

[10] Wang W., Hu S., Gu Y., Yan Y., Stovall D.B., Li D., Sui G. (2020). Human MYC G-quadruplex: From discovery to a cancer therapeutic target. Biochim. Biophys. Acta—Rev. Cancer.

[11] Bahls B., Aljnadi I.M., Emídio R., Mendes E., Paulo A. (2023). G-Quadruplexes in c-MYC Promoter as Targets for Cancer Therapy. Biomedicines.

[12] (2012). Dang C V MYC on the path to cancer. Cell.

[13] Stine Z.E., Walton Z.E., Altman B.J., Hsieh A.L., Dang C.V. (2015). MYC, Metabolism, and Cancer. Cancer Discov..

[14] Kontomanolis E.N., Koutras A., Syllaios A., Sschiza D., Mastoraki A., Garmpis N., DIiakosavvas M., Angelou K., Tsatsaris G., Pagkalos A. (2020). Role of Oncogenes and Tumor-suppressor Genes in Carcinogenesis: A Review. Anticancer Res..

[15] Zakiryanova G.K., Wheeler S., Shurin M.R. (2018). Oncogenes in immune cells as potential therapeutic targets. ImmunoTargets Ther..

[16] Hurley L.H., Wheelhouse R.T., Sun D., Kerwin S.M., Salazar M., Fedoroff O.Y., Han F.X., Han H., Izbicka E., Von Hoff D.D. (2000). G-quadruplexes as targets for drug design. Pharmacol. Ther..

[17] Oganesian L., Bryan T.M. (2007). Physiological relevance of telomeric G-quadruplex formation: A potential drug target. BioEssays.

[18] Zhang S., Wu Y., Zhang W. (2014). G-quadruplex structures and their interaction diversity with ligands. ChemMedChem.

[19] Chen C., Wang Q., Liu J., Hao Y., Tan Z. (2011). Contribution of Telomere G-Quadruplex Stabilization to the Inhibition of Telomerase-Mediated Telomere Extension by Chemical Ligands. J. Am. Chem. Soc..

[20] Phan A.T. (2010). Human telomeric G-quadruplex: Structures of DNA and RNA sequences. FEBS J..

[21] Cogoi S., Xodo L.E. (2006). G-quadruplex formation within the promoter of the KRAS proto-oncogene and its effect on transcription. Nucleic Acids Res..

[22] Kim N. (2019). The Interplay between G-quadruplex and Transcription. Curr. Med. Chem..

[23] Fletcher T.M., Sun D., Salazar M., Hurley L.H. (1998). Effect of DNA secondary structure on human telomerase activity. Biochemistry.

[24] Siddiqui-Jain A., Grand C.L., Bearss D.J., Hurley L.H. (2002). Direct evidence for a G-quadruplex in a promoter region and its targeting with a small molecule to repress c- MYC transcription. Proc. Natl. Acad. Sci. USA.

[25] Wang R., Zhang B., Liang Z., He Y., Wang Z., Ma X., Yao X., Sun J., Wang J. (2019). Insights into rapid photodynamic inactivation mechanism of Staphylococcus aureus via rational design of multifunctional nitrogen-rich carbon-coated bismuth/cobalt nanoparticles. Appl. Catal. B.

[26] Kumari S., Bugaut A., Huppert J.L., Balasubramanian S. (2007). An RNA G-quadruplex in the 5′ UTR of the NRAS proto-oncogene modulates translation. Nat. Chem. Biol..

[27] Reed J.E., Neidle S., Vilar R. (2007). Stabilisation of human telomeric quadruplex DNA and inhibition of telomerase by a platinum–phenanthroline complex. Chem. Commun..

[28] Fouquerel E., Parikh D., Opresko P. (2016). DNA damage processing at telomeres: The ends justify the means. DNA Repair.

[29] Karlseder J. (2003). Telomere repeat binding factors: Keeping the ends in check. Cancer Lett..

[30] Lemarteleur T., Gomez D., Paterski R., Mandine E., Mailliet P., Riou J.F. (2004). Stabilization of the c-myc gene promoter quadruplex by specific ligands’ inhibitors of telomerase. Biochem. Biophys. Res. Commun..

[31] Hassani L., Fazeli Z., Safaei E., Rastegar H., Akbari M. (2014). A spectroscopic investigation of the interaction between c-MYC DNA and tetrapyridinoporphyrazinatozinc(II). J. Biol. Phys..

[32] Ramos C.I.V., Almodôvar V.A.S., Candeias N.R., Santos T., Cruz C., Neves M.G.P.M.S., Tomé A.C. (2022). Diketopyrrolo[3,4-c]pyrrole derivative as a promising ligand for the stabilization of G-quadruplex DNA structures. Bioorg. Chem..

[33] Bidzinska J., Cimino-Reale G., Zaffaroni N., Folini M. (2013). G-quadruplex structures in the human genome as novel therapeutic targets. Molecules.

[34] Kerkour A., Marquevielle J., Ivashchenko S., Yatsunyk L.A., Mergny J.-L., Salgado G.F. (2017). High-resolution three-dimensional NMR structure of the KRAS proto-oncogene promoter reveals key features of a G-quadruplex involved in transcriptional regulation. J. Biol. Chem..

[35] Rapozzi V. (1964). Medicinal Chemistry: A Series of Monographs. Med. Chem..

[36] Müller S., Rodriguez R. (2014). G-quadruplex interacting small molecules and drugs: From bench toward bedside. Expert Rev. Clin. Pharmacol..

[37] Tan J.-H., Gu L.-Q., Wu J.-Y. (2008). Design of selective G-quadruplex ligands as potential anticancer agents. Mini-reviews Med. Chem..

[38] Deiana M., Matczyszyn K., Massin J., Olesiak-Banska J., Andraud C., Samoc M. (2015). Interactions of isophorone derivatives with DNA: Spectroscopic studies. PLoS ONE.

[39] Monchaud D., Granzhan A., Saettel N., Guédin A., Mergny J.-L., Teulade-Fichou M.-P. (2010). “One Ring to Bind Them All”—Part I: The Efficiency of the Macrocyclic Scaffold for G-Quadruplex DNA Recognition. J. Nucleic Acids.

[40] Duarte A.R., Cadoni E., Ressurreição A.S., Moreira R., Paulo A. (2018). Design of Modular G-quadruplex Ligands. ChemMedChem.

[41] Ramos C.I.V., Monteiro A.R., Moura N.M.M., Faustino M.A.F., Trindade T., Neves M.G.P.M.S. (2021). The interactions of h2tmpyp, analogues and its metal complexes with dna g-quadruplexes—An overview. Biomolecules.

[42] Ramos C.I.V., Almeida S.P., Lourenço L.M.O., Pereira P.M.R., Fernandes R., Faustino M.A.F., Tomé J.P.C., Carvalho J., Cruz C., Neves M.G.P.M.S. (2019). Multicharged Phthalocyanines as Selective Ligands for G-Quadruplex DNA Structures. Molecules.

[43] Taquet J., Frochot C., Manneville V., Barberi-Heyob M. (2007). Phthalocyanines Covalently Bound to Biomolecules for a Targeted Photodynamic Therapy. Curr. Med. Chem..

[44] Ramos C.I.V., Tomé J.P.C., Santana-Marques M.G. (2012). Charge and substituent effects on the stability of porphyrin/G-quadruplex adducts. J. Mass Spectrom..

[45] Moura N.M.M., Cavaleiro J.A.S., Neves M.G.P.M.S., Ramos C.I.V. (2023). opp-Dibenzoporphyrin Pyridinium Derivatives as Potential G-Quadruplex DNA Ligands. Molecules.

[46] Monteiro A.R., Ramos C.I.V., Lourenço L.M.O., Fateixa S., Rodrigues J., Neves M.G.P.M.S., Trindade T. (2022). Interfacial assembly of zinc(II) phthalocyanines on graphene oxide (GO): Stable “turn-off-on” nanoplatforms to detect G-quadruplexes (G4). J. Colloid Interface Sci..

[47] Monteiro A.R., Ramos C.I.V., Fateixa S., Moura N.M.M., Neves M.G.P.M.S., Trindade T. (2018). Hybrids Based on Graphene Oxide and Porphyrin as Tools for Detection and Stabilization of DNA G-Quadruplexes. ACS Omega.

[48] Menilli L., Monteiro A.R., Lazzarotto S., Morais F.M.P., Gomes A.T.P.C., Moura N.M.M., Fateixa S., Faustino M.A.F., Neves M.G.P.M.S., Trindade T. (2021). Graphene oxide and graphene quantum dots as delivery systems of cationic porphyrins: Photo-antiproliferative activity evaluation towards t24 human bladder cancer cells. Pharmaceutics.

[49] Sun D., Thompson B., Cathers B.E., Salazar M., Kerwin S.M., Trent J.O., Jenkins T.C., Neidle S., Hurley L.H. (1997). Inhibition of human telomerase by a G-Quadruplex-Interactive compound. J. Med. Chem..

[50] Romera C., Bombarde O., Bonnet R., Gomez D., Dumy P., Calsou P., Gwan J.F., Lin J.H., Defrancq E., Pratviel G. (2011). Improvement of porphyrins for G-quadruplex DNA targeting. Biochimie.

[51] Monchaud D., Teulade-Fichou M.P. (2008). A hitchhiker’s guide to G-quadruplex ligands. Org. Biomol. Chem..

[52] Mishra S., Kota S., Chaudhary R., Misra H.S. (2021). Guanine quadruplexes and their roles in molecular processes. Crit. Rev. Biochem. Mol. Biol..

[53] Yao X., Song D., Qin T., Yang C., Yu Z., Li X., Liu K., Su H. (2017). Interaction between G-Quadruplex and Zinc Cationic Porphyrin: The Role of the Axial Water. Sci. Rep..

[54] Dupont J.I., Henderson K.L., Metz A., Le V.H., Emerson J.P., Lewis E.A. (2016). Calorimetric and spectroscopic investigations of the binding of metallated porphyrins to G-quadruplex DNA. Biochim. Biophys. Acta—Gen. Subj..

[55] Boschi E., Davis S., Taylor S., Butterworth A., Chirayath L.A., Purohit V., Siegel L.K., Buenaventura J., Sheriff A.H., Jin R. (2016). Interaction of a cationic porphyrin and its metal derivatives with G-quadruplex DNA. J. Phys. Chem. B.

[56] Bhattacharjee A.J., Ahluwalia K., Taylor S., Jin O., Nicoludis J.M., Buscaglia R., Brad Chaires J., Kornfilt D.J.P., Marquardt D.G.S., Yatsunyk L.A. (2011). Induction of G-quadruplex DNA structure by Zn(II) 5,10,15,20-tetrakis(N- methyl-4-pyridyl)porphyrin. Biochimie.

[57] Medici S., Peana M., Crisponi G., Nurchi V.M., Lachowicz J.I., Remelli M., Zoroddu M.A. (2016). Silver coordination compounds: A new horizon in medicine. Coord. Chem. Rev..

[58] Jung W.K., Koo H.C., Kim K.W., Shin S., Kim S.H., Park Y.H. (2008). Antibacterial Activity and Mechanism of Action of the Silver Ion in Staphylococcus aureus and Escherichia coli. Appl. Environ. Microbiol..

[59] Gao L.-X., Wu D.-Z., Bigdeli F., Miao Q., Hu M.-L., Morsali A. (2020). Synthesis of a new binuclear silver(I) complex with the ability to interact with DNA molecule. Mater. Lett..

[60] Gil-Moles M., Olmos M.E., Monge M., Beltrán-Visiedo M., Marzo I., López-de-Luzuriaga J.M., Concepción Gimeno M. (2023). Silver-Based Terpyridine Complexes as Antitumor Agents. Chem.—Eur. J..

[61] Moura N.M.M., Guedes S., Salvador D., Oliveira H., Alves M.Q., Paradis N., Wu C., Neves M.G.P.M.S., Ramos C.I.V. (2024). Oncogenic and telomeric G-quadruplexes: Targets for porphyrin-triphenylphosphonium conjugates. Int. J. Biol. Macromol..

[62] Kawauchi K., Urano R., Kinoshita N., Kuwamoto S., Torii T., Hashimoto Y., Taniguchi S., Tsuruta M., Miyoshi D. (2020). Photosensitizers based on g-quadruplex ligand for cancer photodynamic therapy. Genes.

[63] Cheng M., Cui Y.-X., Wang J., Zhang J., Zhu L.-N., Kong D.-M. (2019). G-Quadruplex/Porphyrin Composite Photosensitizer: A Facile Way to Promote Absorption Redshift and Photodynamic Therapy Efficacy. ACS Appl. Mater. Interfaces.

[64] Slomp A.M., Barreira S.M.W., Carrenho L.Z.B., Vandresen C.C., Zattoni I.F., Ló S.M.S., Dallagnol J.C.C., Ducatti D.R.B., Orsato A., Duarte M.E.R. (2017). Photodynamic effect of meso-(aryl)porphyrins and meso-(1-methyl-4-pyridinium)porphyrins on HaCaT keratinocytes. Bioorg. Med. Chem. Lett..

[65] Taylor R.S., Ramirez R.D., Ogoshi M., Chaffins M., Piatyszek M.A., Shay J.W. (1996). Detection of telomerase activity in malignant and nonmalignant skin conditions. J. Investig. Dermatol. Apr..

[66] Lago S., Nadai M., Cernilogar F.M., Kazerani M., Domíniguez Moreno H., Schotta G., Richter S.N. (2021). Promoter G-quadruplexes and transcription factors cooperate to shape the cell type-specific transcriptome. Nat. Commun..

[67] Pavez Lorie E., Stricker N., Plitta-Michalak B., Chen I.-P., Volkmer B., Greinert R., Jauch A., Boukamp P., Rapp A. (2020). Characterisation of the novel spontaneously immortalized and invasively growing human skin keratinocyte line HaSKpw. Sci. Rep..

[68] Harle-Bachor C., Boukamp P. (1996). Telomerase activity in the regenerative basal layer of the epidermis inhuman skin and in immortal and carcinoma-derived skin keratinocytes. Proc. Natl. Acad. Sci. USA.

[69] Fusenig N.E., Boukamp P. (1998). Multiple stages and genetic alterations in immortalization, malignant transformation, and tumor progression of human skin keratinocytes. Mol. Carcinog..

[70] Murat P., Singh Y., Defrancq E. (2011). Methods for investigating G-quadruplex DNA/ligand interactions. Chem. Soc. Rev..

[71] Jaumot J., Gargallo R. (2012). Experimental methods for studying the interactions between G-quadruplex structures and ligands. Curr. Pharm. Des..

[72] Shi D.F., Wheelhouse R.T., Sun D., Hurley L.H. (2001). Quadruplex-interactive agents as telomerase inhibitors: Synthesis of porphyrins and structure-activity relationship for the inhibition of telomerase. J. Med. Chem..

[73] Wei C.Y., Jia G.Q., Yuan J.L., Feng Z.C., Li C. (2006). A spectroscopic study on the interactions of porphyrin with G- quadruplex DNAs. Biochemistry.

[74] Bhattacharjee S., Sengupta P.K., Bhowmik S. (2017). Exploring the preferential interaction of quercetin with VEGF promoter G-quadruplex DNA and construction of a pH-dependent DNA-based logic gate. RSC Adv..

[75] Largy E., Granzhan A., Hamon F., Verga D., Teulade-Fichou M.P. (2013). Visualizing the Quadruplex: From Fluorescent Ligands to Light-Up Probes. Top. Curr. Chem..

[76] Prinz H. (2010). Hill coefficients, dose–response curves and allosteric mechanisms. J. Chem. Biol..

[77] Han H., Hurley L.H. (2000). G-quadruplex DNA: A potential target for anti-cancer drug design. Trends Pharmacol. Sci..

[78] Largy E., Hamon F., Teulade-Fichou M.P. (2011). Development of a high-throughput G4-FID assay for screening and evaluation of small molecules binding quadruplex nucleic acid structures. Anal. Bioanal. Chem..

[79] Monchaud D., Teulade-Fichou M.P., Baumann P. (2009). G4-FID: A Fluorescent DNA Probe Displacement Assay for Rapid Evaluation of Quadruplex Ligands. G-Quadruplex DNA—Methods and Protocols.

[80] Spiller W., Kliesch H., Wöhrle D., Hackbarth S., Röder B., Schnurpfeil G. (1998). Singlet Oxygen Quantum Yields of Different Photosensitizers in Polar Solvents and Micellar Solutions. J. Porphyr. Phthalocyanines.

[81] Spesia M.B., Milanesio M.E., Durantini E.N. (2008). Synthesis, properties and photodynamic inactivation of Escherichia coli by novel cationic fullerene C60 derivatives. Eur. J. Med. Chem..

[82] Zimcik P., Miletin M., Radilova H., Novakova V., Kopecky K., Svec J., Rudolf E. (2010). Synthesis, Properties and In Vitro Photodynamic Activity of Water-soluble Azaphthalocyanines and Azanaphthalocyanines. Photochem. Photobiol..

[83] Rajora M.A., Lou J.W.H., Zheng G. (2017). Advancing porphyrin’s biomedical utility via supramolecular chemistry. Chem. Soc. Rev..

[84] Dąbrowski J.M., Pucelik B., Regiel-Futyra A., Brindell M., Mazuryk O., Kyzioł A., Stochel G., Macyk W., Arnaut L.G. (2016). Engineering of relevant photodynamic processes through structural modifications of metallotetrapyrrolic photosensitizers. Coord. Chem. Rev..

[85] del Toro M., Gargallo R., Eritja R., Jaumot J. (2008). Study of the interaction between the G-quadruplex-forming thrombin-binding aptamer and the porphyrin 5,10,15,20-tetrakis-(N-methyl-4-pyridyl)-21,23H-porphyrin tetratosylate. Anal. Biochem..

[86] Litovchick A., Tian X., Monteiro M.I., Kennedy K.M., Guié M.-A., Centrella P., Zhang Y., Clark M.A., Keefe A.D. (2019). Novel Nucleic Acid Binding Small Molecules Discovered Using DNA-Encoded Chemistry. Molecules.

[87] Sun D., Hurley L.H. (2010). Biochemical techniques for the characterization of Gquadruplex structures: EMSA, DMS footprinting, and DNA polymerase stop assay. Methods Mol. Biol..

[88] Voytik-Harbin S.L., Brightman A.O., Waisner B., Lamar C.H., Badylak S.F. (1998). Application and evaluation of the alamarblue assay for cell growth and survival of fibroblasts. Vitr. Cell. Dev. Biol.—Anim..

[89] Rampersad S.N. (2012). Multiple Applications of Alamar Blue as an Indicator of Metabolic Function and Cellular Health in Cell Viability Bioassays. Sensors.

[90] Nakayama G.R., Caton M.C., Nova M.P., Parandoosh Z. (1997). Assessment of the Alamar Blue assay for cellular growth and viability in vitro. J. Immunol. Methods.

[91] Vallejo M.C.S., Reis M.J.A., Pereira A.M.V.M., Serra V.V., Cavaleiro J.A.S., Moura N.M.M., Neves M.G.P.M.S. (2021). Merging pyridine(s) with porphyrins and analogues: An overview of synthetic approaches. Dye. Pigment..

[92] Nagesh N., Sharma V.K., Ganesh Kumar A., Lewis E.A. (2010). Effect of ionic strength on porphyrin drugs interaction with quadruplex DNA formed by the promoter region of C-myc and Bcl2 oncogenes. J. Nucleic Acids.

[93] Lakowicz J.R., Lakowicz J.R. (2006). Principles of Fluorescence Spectroscopy.

[94] van de Weert M., Stella L. (2011). Fluorescence quenching and ligand binding: A critical discussion of a popular methodology. J. Mol. Struct..

