# Supplementary material for: Is Silver a Precious Metal for G-Quadruplex Stabilization Mediated by Porphyrins?
Source: Int J Mol Sci. 2024 Dec 18;25(24):13556. doi: 10.3390/ijms252413556 (PMC11678824; doi:10.3390/ijms252413556)
Supplement: Supplementary file 1 [file ijms-25-13556-s001.zip › ijms-3300702-supplementary.pdf]

Is Silver a Precious Metal for G-Quadruplex Stabilization Mediated by Porphyrins?

Nuno M. M. Moura [1], Sofia Guedes [1], Diana Salvador [2,3], Helena Oliveira [2], M. Graça P. M. S. Neves [1,*] and Catarina I. V. Ramos [1,*]

[1] LAQV-REQUIMTE, Department of Chemistry, University of Aveiro, 3810-193 Aveiro, Portugal; nmoura@ua.pt (N.M.M.M.); sguedes@ua.pt (S.G.)
[2] CESAM-Centre for Environmental and Marine Studies, Department of Biology and CESAM, University of Aveiro, 3810-193 Aveiro, Portugal; diana.s@ua.pt (D.S.); holiveira@ua.pt (H.O.)
[3] CICECO, Aveiro Institute of Materials, Department of Chemistry, University of Aveiro, 3810-193 Aveiro, Portugal
* Correspondence: gneves@ua.pt (M.G.P.M.S.N.); c.ramos@ua.pt (C.I.V.R.)

Supplementary Materials

1. Oligonucleotide sequences

Table S1. DNA sequences

	Oligonucleotide	Sequence (from 5′-….- 3′)	Description
**DNA titrations**	**G4 Tel**	A**GGG**TTA**GGG**TTA**GGG**TTA**GGG**	Human telomeric repeat
	MYC	TGA**GGG**T**GGG**TA**GGG**T**GGG**TAA	Oncogene promoter
	KRAS	A**GGGCGG**TGT**GGG**AATA**GGG**AA	Oncogene promoter
	**ds26**	CAATCGGATCGAATTCGATCCGATTG	Double-stranded DNA
**PCR- Stop Assay**	**Pu28**	TAATACGACTCACTATAGCAATTGCGTG	Primer
	**Pu77**	TCCAACTATGTATACT**GGGGAGGGTGGG**<br>GA**GGGTGGGG**AAGGTTAGCGGCACGCA<br>ATTGCTATAGTGAGTCGTATTA	*MYC* template
	**Pu77 mut**	TCCAACTATGTATACTAAGGAAAGTAA<br>GGAAAGTAAGGAAGGTTAGCGGCACG<br>CAATTGCTATAGTGAGTCGTATTA	Mutant *MYC* template

2. Results

2.1. *UV-Vis spectroscopy*

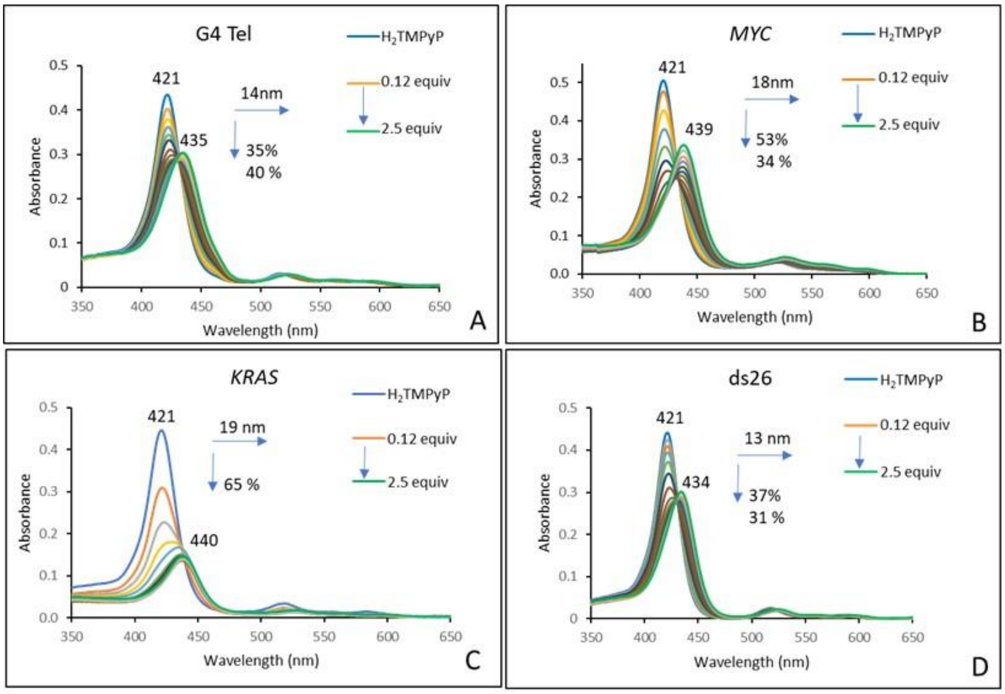

Figure S1 - UV-vis spectra obtained from the titration of **H_2TMPyP** ($2x10^{-6}$ M), in PBS (10 mM KH_2PO_4, 10 mM K_2HPO_4 and 100 mM KCl; pH 6.8), with increasing amounts (0.125 to 2.5 equiv.) of A - G4 Tel; B - *MYC*; C- *KRAS*; D - ds26.

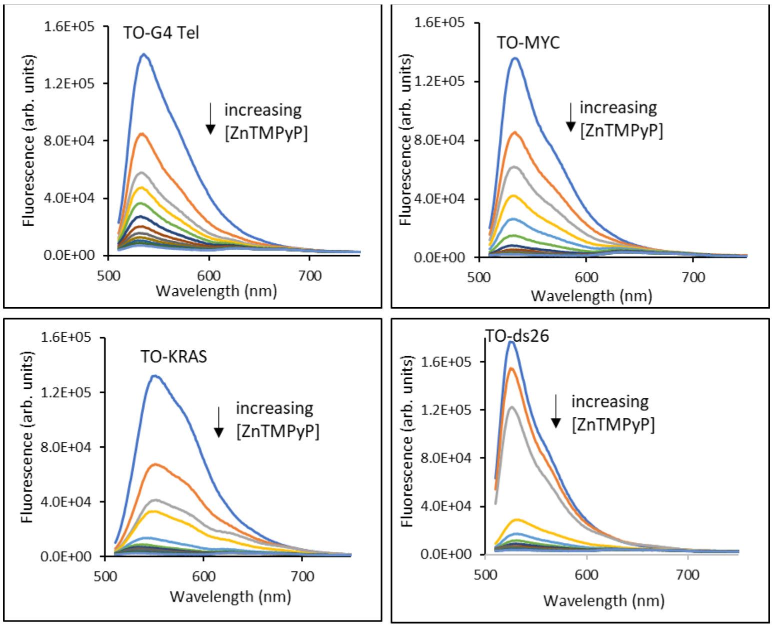

Figure S2 – FID spectra obtained from the titration of the adduct solutions TO (3.5 μM):DNA (1 μM) prepared in PBS (10 mM KH_2PO_4, 10 mM K_2HPO_4 and 100 mM KCl; pH 6.8) with increasing amounts (0.125 to 2.5 equiv.) of **ZnTMPyP**, prepared in PBS (10 mM KH_2PO_4, 10 mM K_2HPO_4 and 100 mM KCl; pH 6.8

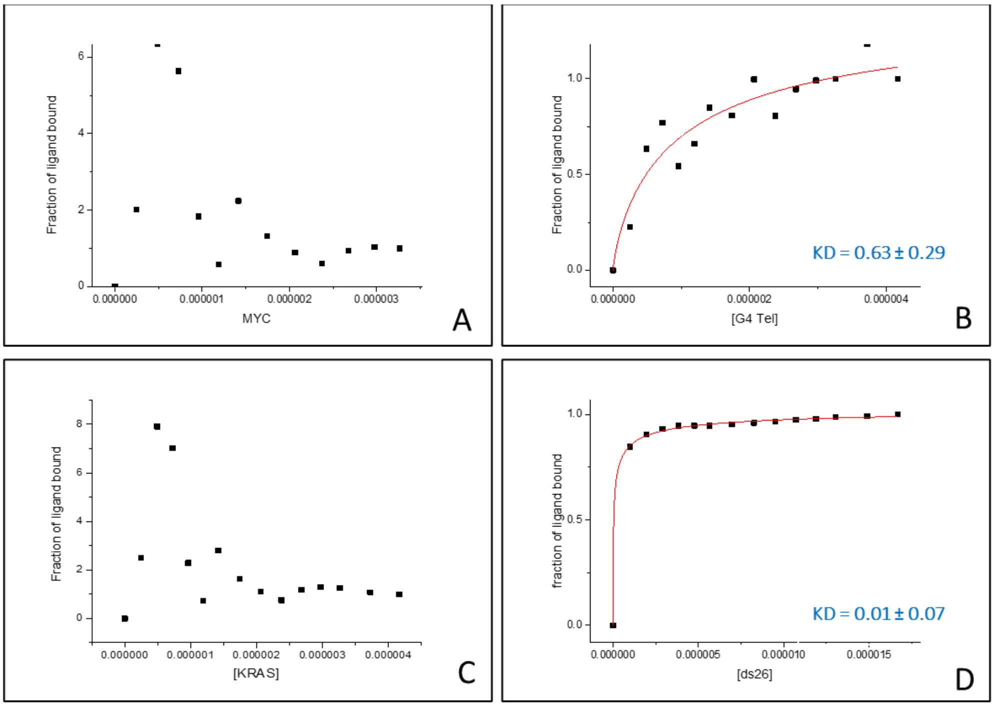

Figure S3 – Plots of alpha versus [DNA] obtained from fluorescence titration of **AgTMPyP** (2 μM) and A—*MYC*, B—G4 Tel, C—*KRAS*, and D—ds26;

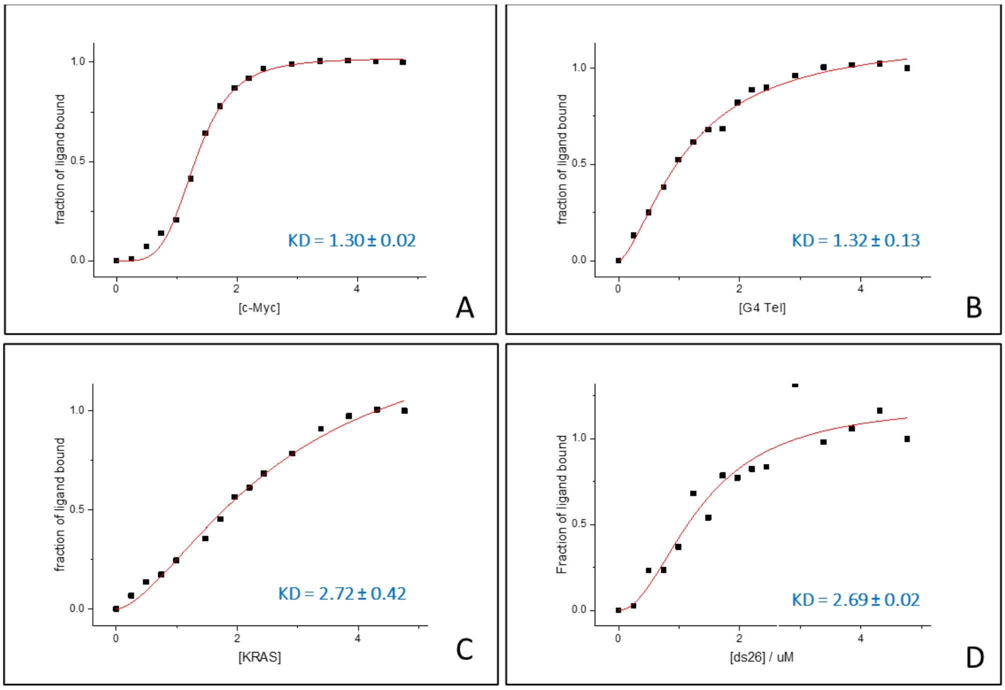

Figure S4 – Plots of alpha versus [DNA] obtained from fluorescence titration of **ZnTMPyP** (2 μM) and A—*MYC*, B—G4 Tel, C—*KRAS*, and D—ds26;

3. Detailed methodologies

3.1. Synthesis of the Ag^{II}TMPyP complex

The Ag(II) complex of TMPyP was prepared by modification of a procedure previously described in the literature [1]. TMPyP (15 mg, 2.4 x 10^{-5} mol) was dissolved in DMF and $AgNO_3$ (4.1 mg, 2.4 x 10-5 mol) was added to the solution and the mixture was stirred at 80 °C for 24 h. After, the metalloporphyrin was precipitated with diethyl ether and the resulting solid was washed with a diethyl ether/propan-1-ol (2:1) mixture. The desired complex was dried in the oven at 40 °C for 24 h. The structure of the AgIITMPyP was confirmed by mass spectrometry; the mass spectrum displays a m/z peak at 196.3 corresponding to the [M]4+ ion with *m*/*z* 196.32 (Figure S1).

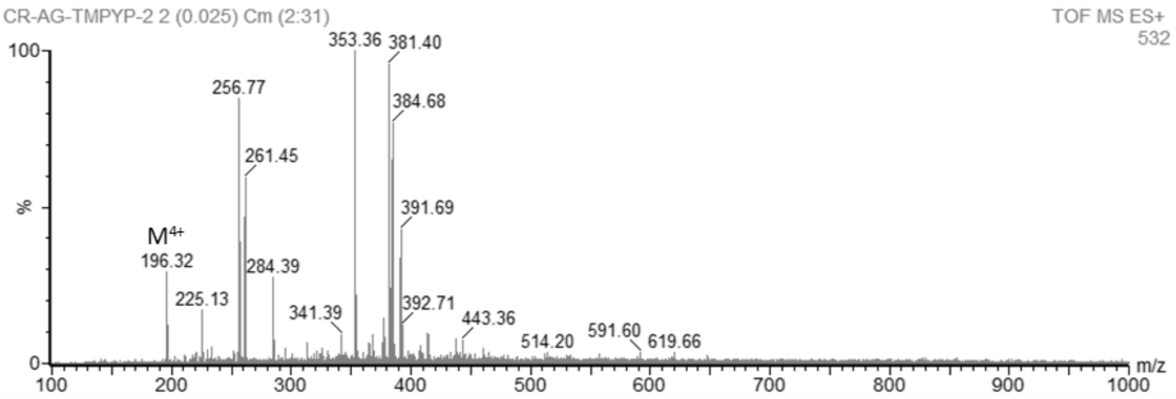

Figure S5 – Mass spectrum of the **Ag^{II}TMPyP** derivative.

3.2. UV-Vis spectroscopy

UVVis absorption spectra were recorded in a Shimadzu UV-2501-PC spectrophotometer in the range of 350-750 nm, using a 1 cm length quartz cuvette; a Huber Compatible Control CC1 at 25 °C was used for temperature control. The titrations were performed by adding to the $2x10^{-6}$ M porphyrin solutions increasing amounts of each oligonucleotide solutions (0 to 2.5 equiv.) and ended when, during three successive additions no meaningful absorbance changes were observed [80]. Blank assays were carried out by adding small amounts of PBS buffer to ligand solution. The dilution effects in the spectra were mathematically corrected using the formula Abscor =[(Vi + Vad)/Vi]*Abs, where Abscor is the corrected absorbance, Vi is the initial volume of the ligand in the cuvette, Vad is the volume of DNA added and Abs is the absorbance of each point of the titration. To confirm the results reproducibility, the experiments were performed in triplicate. The equation % hypochromicity = $[(\varepsilon_{free} - \varepsilon_{bound})/\varepsilon_{free}]\times 100$, where ε_{bound} is ($\varepsilon_{bound} = A_{bound}/C_{bound}$) according to Beer's Law and ε_{free} is the obtained extinction coefficient in PBS medium (**H_2TMPyP** ε_{421} = 226.000 $M^{-1}cm^{-1}$, **AgTMPyP** ε_{430} = 136.014 $M^{-1}cm^{-1}$ and **ZnTMPyP** ε_{437} = 225.000 $M^{-1}cm^{-1}$) was used to calculate the percentage of hypochromicity of the absorption bands.

3.3. Fluorescence Spectroscopy

3.3.1. Fluorescence Titrations

The spectra were recorded in a Horiba FluoroMax-4 spectrofluorometer at 25 °C and in a 1 cm path length quartz cuvette. The ligands were excited at maximum absorbance wavelength and the fluorescence emission collected

between 600-825 nm using excitation and emission slits fixed at 5 nm. The apparent dissociation constants (*K*D) between each ligand and DNA structure were assessed by measuring changes in fluorescence during titrating the ligand solution at 2x10^{-6} M with 0 to 2.5 equivalents of the adequate DNA sequence, corresponding to G4 or double-stranded DNA (dsDNA) structures. The following equation was used to convert obtained data into fraction of bound ligand (α) plots:

$$\alpha = \frac{I - I_{\lambda}^{free}}{I_{\lambda}^{bound} - I_{\lambda}^{free}}$$

where I is the fluorescence intensity of each ligand:G4 ratio and I_{bound} and I_{free} are the fluorescence intensity of the fully bound and of the free ligand, respectively. Data points were then fitted to a hyperbolic function using the OriginPro 8 software and K_D values were determined from the following saturation binding model:

$$\alpha = \frac{[DNA]^n}{K_D + [DNA]^n}$$

where α is the fraction of ligand bound, [DNA] is the concentration of the DNA and *n* is the Hill constant which describes cooperativity of ligand binding.

3.3.2. Fluorescence Intercalator Displacement (FID) studies

Stock solutions (35x10^{-6} M) of thiazole orange (TO) and of each oligonucleotide (10x10^{-6} M) were prepared. To the formation of the 1:1 TO-oligonucleotide adducts, the previous solutions were slowly mixed during 10 min in an orbital shaker. The fluorescence of the obtained adducts was confirmed in a FluoroMax-4 spectrofluorometer (Horiba), using the excitation wavelength at 485 nm and analyzing the emission between 510-850 nm; the excitation and emission slits were set at 5 nm.

Concentrations ranging from 0 to 3.2x10^{-6} M of each ligand was then added to each TO-oligonucleotide solution. The resulting fluorescence was then measured and the percentage of displacement calculated using the following equation:

$$\% \text{ displacement} = 100 - \frac{F_A}{F_{A0}} * 100$$

where $F_A = F - F_{H2O}$ and $F_{A0} = F_0 - F_{H2O}$; F is the fluorescence intensity of each sample, F_{H2O} the fluorescence intensity of milli-Q and F_0 the fluorescence from the fluorescent probe bound to DNA without added ligand.

3.4 Cell Viability Assay

HaCaT cells were maintained in high-glucose Dulbecco's Modified Eagle Medium (DMEM), supplemented with 10% non-inactivated foetal bovine serum (FBS), streptomycin (1%) and penicillin (0.6%), 1.5 g/L sodium bicarbonate and $2x10^{-3}$ M of glutamine. The cells were maintained in the culture (cell density 0.5-$0.8x10^6$ cells/mL) at 37 °C and 5% CO_2 in an incubator and, every three days, cells were sub-cultured through trypsinization (0.05% (m/v) EDTA solution and Trypsin/EDTA 0.05% (m/v) in HBSS) as to maintain.

The Alamar Blue Assay was used to determine cell viability in the presence of porphyrins [83]. Dose-response experiments were performed, by exposing cells to a range of porphyrins concentrations, in order to determined EC_{25} was, during 24 h. In brief, HaCaT cells were seeded, in a 96-well plate, at the density of $1.5x10^4$ cells/well and allowed to stabilise and adhere to the plate, for a period of 24 h, in the incubator. Cells were then kept in fresh culture medium, to be used as control, or treated with the free base **H_2TMPyP** or with the silver or zinc(II) mettaloporphyrins**,** in the range of concentrations from 5 to $100x10^{-6}$ M. When the 20 h of incubation period was reaching, cells were treated with $50x10^{-6}$ M resazurin and incubated for 4 h. After 24 h of incubation, resorufin absorbance was read at both 560 and 600 nm and quantified on a plate reader Multiskan GO 1510-00111C (ThermoScientific, Waltham, MA, USA). The assays were done in triplicate.

3.5. Confocal microscopy

HaCaT and MNT-1 cells were grown on glass coverslips in 12 well plates at a density of $3.5x10^4$ cells/mL and then incubated for 24 h at 37 °C and 5% CO_2 for cell attachment. After that, cells were exposed to **H_2TMPyP** and **AgTMPyP** at the concentrations equivalent to IC_{25} for 24 h. Cells were then washed 3 times with warm PBS and fixed with 4% paraformaldehyde in PBS for 10 min at room temperature. After being washed with cold PBS, cells were washed with

abundant Milli-Q water and the coverslips mounted onto glass slides with 4′,6-diamidino-2-phenylindole (DAPI)-containing Vectashield mounting medium (Vector Labs).

Microphotographs were acquired on a Zeiss LSM 880LSM 510 META confocal microscope (Zeiss, Jena, Germany) through a Plan-Neofluor 63x/1.4 oil immersion objective. DAPI fluorescence was collected at 420–480 nm (λ_{exc} = 405 nm) and porphyrin fluorescence at 650–750 nm (λ_{exc} = 405 nm).
